# Online Reduction of Artifacts in EEG of Simultaneous EEG-fMRI Using Reference Layer Adaptive Filtering (RLAF)

**DOI:** 10.1007/s10548-017-0606-7

**Published:** 2017-11-09

**Authors:** David Steyrl, Gunther Krausz, Karl Koschutnig, Günter Edlinger, Gernot R. Müller-Putz

**Affiliations:** 1 Laboratory of Brain-Computer Interfaces, Institute of Neural Engineering, Graz University of Technology, 8010 Graz, Austria; 2 GUGER TECHNOLOGIES OG, Graz, Austria; 3 Department of Psychology, University of Graz, Graz, Austria; 4 BioTechMed-Graz, Graz, Austria

**Keywords:** Simultaneous electroencephalography (EEG) and functional magnetic resonance imaging (fMRI), Artifact reduction, Reference layer adaptive filtering (RLAF), Online processing

## Abstract

Simultaneous electroencephalography (EEG) and functional magnetic resonance imaging (fMRI) allow us to study the active human brain from two perspectives concurrently. Signal processing based artifact reduction techniques are mandatory for this, however, to obtain reasonable EEG quality in simultaneous EEG-fMRI. Current artifact reduction techniques like average artifact subtraction (AAS), typically become less effective when artifact reduction has to be performed on-the-fly. We thus present and evaluate a new technique to improve EEG quality online. This technique adds up with online AAS and combines a prototype EEG-cap for reference recordings of artifacts, with online adaptive filtering and is named reference layer adaptive filtering (RLAF). We found online AAS + RLAF to be highly effective in improving EEG quality. Online AAS + RLAF outperformed online AAS and did so in particular online in terms of the chosen performance metrics, these being specifically alpha rhythm amplitude ratio between closed and opened eyes (3–45% improvement), signal-to-noise-ratio of visual evoked potentials (VEP) (25–63% improvement), and VEPs variability (16–44% improvement). Further, we found that EEG quality after online AAS + RLAF is occasionally even comparable with the offline variant of AAS at a 3T MRI scanner. In conclusion RLAF is a very effective add-on tool to enable high quality EEG in simultaneous EEG-fMRI experiments, even when online artifact reduction is necessary.

## Introduction

Non-invasive neuroimaging techniques offer the unique opportunity to investigate the active human brain without surgery. The two most popular non-invasive neuroimaging techniques are electroencephalography (EEG) and functional magnetic resonance imaging (fMRI) (Michel and Murray 2012; Norris 2006). EEG measures electrical brain activity, whereas fMRI measures blood oxygenation level changes in the brain (Niedermeyer and Lopes da Silva 2005; Ogawa et al. 1990). These two techniques have partly complementary properties. For example, the time resolution of EEG is in the millisecond range, whereas it is in the range of seconds for fMRI. A second example is the spatial resolution of the techniques, which is commonly in the range of millimeters for fMRI and in the range of centimeters for EEG (Laufs 2012; He et al. 2011). The combing of EEG and fMRI was proposed to benefit from the best of both worlds. The combined simultaneous application of these two techniques allows comprehensive studies of the same brain activity from the electrophysiological and from the metabolic/vascular point of view. Examples of such studies include the combined or joint analysis of EEG and fMRI data such as e.g. in EEG-informed fMRI, the localization of transient brain activity, and also the analysis of the interaction of electrophysiology and metabolism (Huster et al. 2012; Uludag and Roebroeck 2014; Debener et al. 2006; Ritter and Villringer 2006). This combination is often referred to as simultaneous EEG-fMRI.

Unfortunately, these two techniques influence each other and deteriorate the data quality of the respective other. The additional EEG equipment inside the MRI scanner interferes with the static magnetic field and with the radio frequency field of the scanner. This interference generates field inhomogeneities and signal losses, which in turn degrade the fMRI data quality. Studies demonstrate that the data quality loss in fMRI varies between negligible and severe, but is never prohibitive (Bonmassar et al. 2010; Luo and Glover 2012; Jorge et al. 2015a). The effect of fMRI data acquisition on the EEG data quality is however critical (Mulert and Lemieux 2010; Mullinger and Bowtell 2011a). Over the past years, a variety of fMRI related artifacts in EEG of simultaneous EEG-fMRI have been described. Below, we give an overview, sorted by the usual magnitudes of the artifacts.

The most prominent artifact is the so-called gradient artifact (GA), sometimes also referred to as the scanner artifact (Allen et al. 2000). It has amplitudes up to 1000 times higher than the EEG (Allen et al. 2000; Mullinger et al. 2011b). The switching of the scanner gradient during fMRI data acquisition causes this artifact by electromagnetic induction in the leads of the EEG electrodes. It repeats whenever a new volume acquisition starts. Although techniques to reduce this artifact are known, it is not possible yet to avoid it completely (Mullinger et al. 2011b; Jorge et al. 2015a; Assecondi et al. 2016). Various signal processing based methods have thus been developed to reduce the impact of this artifact. Average artifact subtraction (AAS) is one of them and probably the most widely used one (Allen et al. 2000). AAS exploits the repetitive and deterministic nature of the GA. A separate artifact template is compiled for each single artifact epoch of each EEG channel by averaging over adjacent epochs. This template is subsequently subtracted from the EEG. By averaging over adjacent artifact epochs, AAS can cope with slow changes of the GA, but not with brisk changes, due to e.g. motion of the study participant. Hence, although AAS reduces the GA largely, residuals of the GA are still present and they can be in the same order of magnitude as the EEG.

Reducing the GA unveils a second artifact, the pulse artifact (PA), which is repetitive with the cardiac-pulse cycle. PA amplitudes have the same order of magnitude as the EEG amplitudes and they increas with the strength of the static magnetic field (Allen et al. 1998; Debener et al. 2007, 2008). The PA itself is mainly caused by motion of EEG electrodes, due to cardiac-pulse driven head nodding and due to expansion of blood vessels below the respective EEG electrode (Bonmassar et al. 2002). A second contributor to this artifact is voltage induction in EEG electrodes due to the acceleration of blood below the electrode. Blood is electrically conductive and therefore surrounded by an electromagnetic field, when accelerated in a static magnetic field. The proportion of this second contributor is relatively small, however, when compared to the first motion related component (Mullinger et al. 2013a). Signal processing based methods are the only option to reduce the artifact and its impact on EEG. AAS is again the most common method to tackle this artifact (Allen et al. 1998). PA epochs are defined by additional electrocardiogram recordings. An individual pulse artifact template per PA epoch and EEG channel is computed by averaging over adjacent PA epochs and subsequently subtracted from the current PA epoch. The cardiac cycle is, however, inherently varying. Hence, the artifact template is only an approximation of the PA and significant PA residuals are often present, particularly at higher static magnetic field strengths of 3T or more. The frequency range of these residuals is usually including the alpha and beta range of EEG and can completely obscure these important brain rhythms.

Other known artifacts in EEG of simultaneous EEG-fMRI are vibration related artifacts like the helium pump artifact (HPA) and the ventilation artifact (VA) (Mullinger et al. 2013b; Nierhaus et al. 2013). Both are caused by MRI scanner systems and are therefore presumably specific to a scanner model. The HPA is mainly generated by vibrations from the cooling system of the MRI scanner, in particular from the helium pump (Nierhaus et al. 2013; Rothlübbers et al. 2014). The VA is caused by vibrations of the patient ventilation system of the MRI scanner (Nierhaus et al. 2013). Both artifacts can be circumnavigated by disabling the systems temporarily. However, both systems are important for a safe and comfortable usage of the MRI scanner and disabling them can be unwanted or not possible. Further, both artifacts are so far not well investigated and artifact reduction techniques are available for the HPA only (Rothlübbers et al. 2014; Kim et al. 2015).

The motion artifact (MA) is another very problematic artifact. It is caused by EEG electrode and cable motion in the static magnetic field of the MRI scanner (Van Der Meer et al. 2010). It is problematic in two senses. First, it is non-repetitive, non-stationary, and typically not predictable. Hence, there is no way to reduce the MA with signal processing based methods that exploit repetitive structures in the artifact. Second, motions change the shape of the GA and the PA. Hence, the AAS approach fails to reduce these artifacts well, since the AAS template is not a good representation of the respective artifact anymore. Many MA reduction techniques have been proposed (Bonmassar et al. 2002; Masterton et al. 2007, Van Der; Meer et al. 2010; Abbott et al. 2014; Jorge et al. 2015b). However, best practice is to prevent them by restricting possible motions of the study participants.

These variety of artifacts in EEG of simultaneous EEG-fMRI recordings and the need to improve EEG quality, have led to the development of many different methods for reducing artifacts. Beside the standard AAS approach, particularly the optimal basis set (OBS) approach and the independent component analysis (ICA) approach are frequently used (Niazy et al. 2005; Srivastava et al. 2005; Briselli et al. 2006; Mantini et al. 2007; Ritter et al. 2007; Vanderperren et al. 2010; Abreu et al. 2016). Other methods, for example based on beam former, singular value decomposition, linear predictors, independent vector analysis and dictionary learning, were introduced too and can outperform the aforementioned methods under certain conditions (Brookes et al. 2008; Liu et al. 2012; Ferdowsi et al. 2013; Acharjee et al. 2015; Abolghasemi and Ferdowsi 2015).

Apart from the interest in techniques that improve EEG quality of simultaneous EEG-fMRI in general, there is also growing interest in special techniques that reduce the above-mentioned artifacts on-the-fly. Specifically, in order to carry out experiments, where online processing of the measured data is required. In this context, online processing of data means timely signal processing without knowing the future data, hence signal processing that relies on the past data only, also known as causal signal processing. Some examples for experiments of this kind are: (1) Triggering visual stimulation depending on ongoing EEG and investigating the effects with fMRI (Becker et al. 2011). (2) Locating cerebral generators of epilepsy spikes online (Gotman et al. 2006). (3) Investigating brain activity with fMRI during the use of EEG neurofeedback (Zotev et al. 2014; Zich et al. 2014, 2015). (4) The construction of a new type of brain-computer interfaces (BCIs) that rely on the online feedback of two neuroimaging modalities, hence simultaneous EEG and fMRI feedback, to generate control signals for an application or for the paradigm itself (Brunner et al. 2015; Mano et al. 2017; Perronnet et al. 2017). Unfortunately, most of the MRI artifact reduction methods rely on non-causal signal processing, hence knowledge of upcoming data is required and they can therefore only be applied offline, after the experiment. This situation led to the development of online applicable artifact reduction techniques. Brain Products (Brain Products GmbH, Gilching, Germany) provide an online version of AAS for GA and PA reduction in their commercial RecView tool. Other online artifact reduction methods, for example based on windowed versions of OBS and ICA, have also been developed (Wu et al. 2016; Mayeli et al. 2016; Wen et al. 2016).

The EEG data quality of simultaneous EEG-fMRI is often mediocre. For example, Zich et al. carried out a BCI experiment based on the classification of sensorimotor rhythms and they report a drop in average classification accuracy by approximately 10% when moving from outside the scanner to inside the scanner (Zich et al. 2015). In a similar experiment with a single participant, we found the classification accuracy to be 22% lower inside the scanner compared to outside the scanner (Steyrl et al. 2013). One reason for the poorer EEG data quality can be found in the artifact reduction methods. Both studies used AAS and as mentioned above, AAS is susceptible to brisk artifact changes and inherently varying artifacts. Moreover, AAS also depends on reliable detection of artifact periods. And naturally, AAS is only able to reduce repetitive artifacts such as the GA and the PA. Unfortunately, switching to another artifact reduction method is not necessarily a solution. The limitations of AAS also hold for the OBS method. ICA based methods on the other hand are partly able to reduce other artifact types too, however, they rely on the basic assumption that artifacts, or components, are stationary in space, which is particularly violated for PAs (Debener et al. 2007).

We recently presented a new add on technique for artifact reduction in EEG of simultaneous EEG-fMRI, which uses a completely different approach. This technique is based on the idea of recording artifacts independently of, but simultaneously with EEG, at a reference layer that is isolated from the scalp. Adaptive filters use those independent reference recordings to reduce the artifacts in the EEG. This technique is therefore named reference layer adaptive filtering (RLAF) (Steyrl et al. 2015, 2017; Chowdhury et al. 2014; Dunseath and Alden 2009; McGlone et al. 2009). In our previous works, we presented a reusable EEG-cap prototype that is equipped with a saline-water based reference layer to allow the aforementioned independent reference recordings (Steyrl et al. 2017). We showed that RLAF is most effective when artifacts have already been reduced using another technique such as AAS in a pre-processing step. We reported on artifact reduction results of data recorded at a spherical fMRI phantom, as well as on artifact reduction results of human EEG (Steyrl et al. 2015, 2017). Our results demonstrate that RLAF tackles all artifacts that occur, as long as they are represented in the reference layer, which leads to a substantially improved EEG quality compared to predecessor techniques (Steyrl et al. 2017).

In this work, we introduce RLAF for the online artifact reduction in EEG of simultaneous EEG-fMRI. As in our previous work, RLAF is applied as an add on after AAS and in this case after online AAS. The evaluation of EEG artifact reduction techniques is generally tricky, since a basic truth in this issue remains an unknown factor. Several suggestions for evaluation strategies have been made, but despite this a gold standard has not emerged yet. For this work, we decided to focus on the evaluation of what can be assumed as the best known and most widely analyzed EEG phenomena. We analyze alpha rhythm amplitude differences between opened and closed eyes, and visual evoked potentials (VEP). We compare the online version of AAS + RLAF with: the online version of AAS, the offline version of AAS, and EEG recorded outside the MRI scanner.

## Materials and Methods

### Participants

The experiment was performed in accordance with the Declaration of Helsinki and was approved by the local ethics committee. Seven participants (all male, students, age 21–26 years) volunteered in this experiment. One was excluded, because he felt uncomfortable inside the scanner and aborted the experiment. Participants had medical histories free of neurological abnormalities and gave written informed consent for participation before the experiment. They received a monetary compensation of 20 €.

### Experiment Description

The aim of the present work was to record EEG, specifically alpha rhythm amplitude differences and evoked brain responses and to compare those from measurements inside and outside the MRI scanner. Hence, each participant performed the experiment twice. First, recordings were performed outside the MRI scanner, in the room where the EEG cap was prepared and then a second time inside the MRI scanner. We used a modified version of the experiment in our last RLAF work (Steyrl et al. 2017). The experiment itself was divided into two parts. During the first part, evoked brain responses were recorded. Participants had their eyes opened and were looking at a computer monitor, where a checkerboard was presented. The checkerboard had 8 × 8 black and white square fields with a small red dot in the center. The black and white fields were inverted every 0.5–0.6 s to trigger visual evoked potentials (VEP). 600 VEPs were collected per experiment. In the second part of the experiment, participants closed their eyes and were instructed to relax, but not to fall asleep, to provoke changes in the alpha rhythm. The experiments outside and inside the scanner differed in three points: (1) Outside the scanner, participants were upright sitting in a chair. Inside the scanner, participants were lying in supine position. (2) The distance between monitor and eyes was about 1 m in the experiments outside the scanner (visual angle 20°), and approximately 2.5 m in the experiments inside the scanner (visible angle 15°). (3) Outside the scanner, the environment was quiet. Inside the scanner, we used earplugs to reduce the scanner noise. One experiment lasted in total about 12 min with approximately 6 min opened eyes and 6 min closed eyes. The overall time per participant was about 2 h with 20 min for instructions and information, 40 min cap preparation and testing, 12 min experiment outside, 20 min preparation inside scanner, 10 min testing inside scanner, 12 min experiment inside scanner, and 5 min for removing the equipment from the participant.

### Reference Layer Cap Prototype

**Fig. 1:**
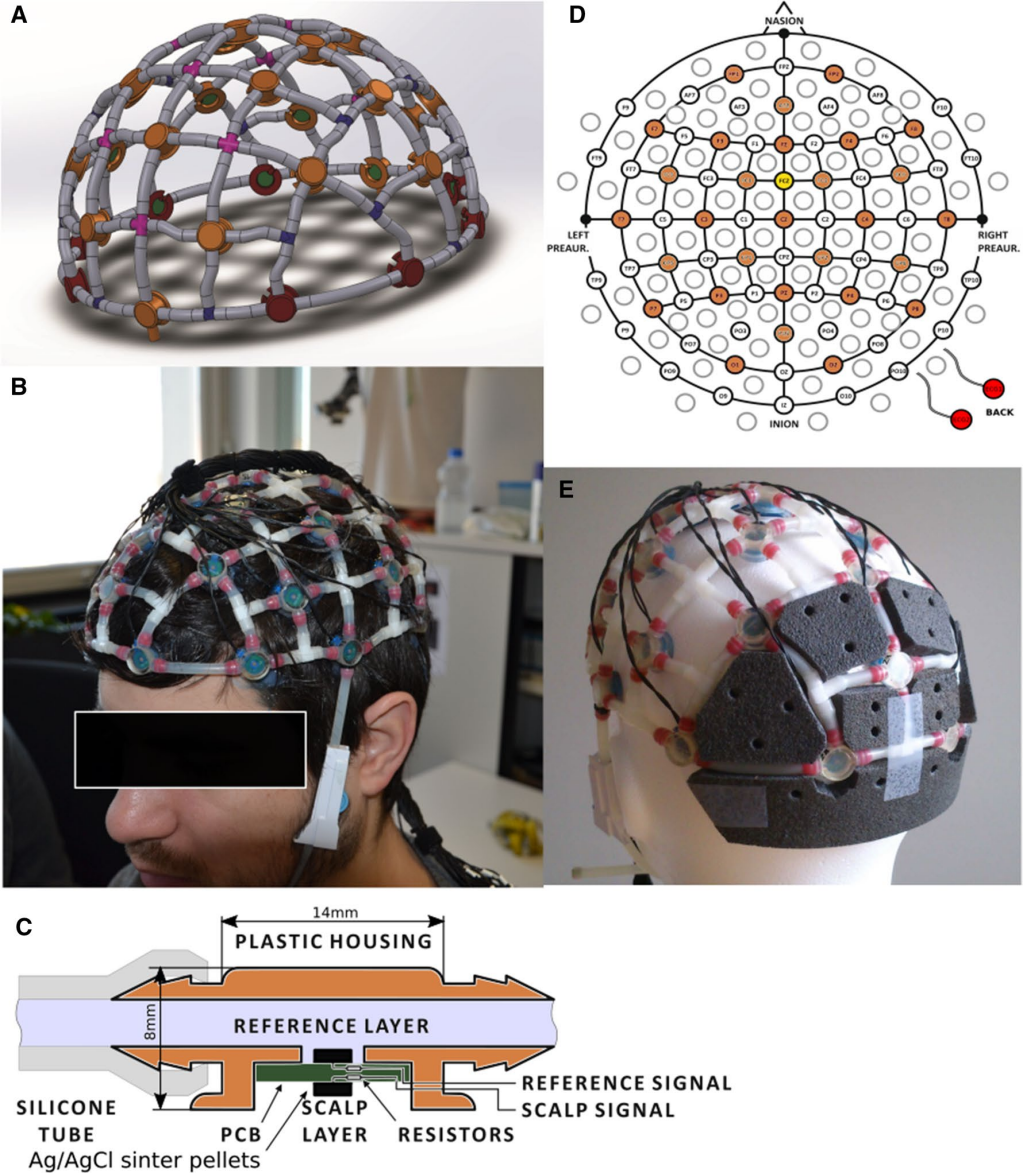
Reference layer cap prototype. **a** Rendering of the cap prototype, **b** cap mounted on a head, **c** schematics of a reference layer electrode pair, **d** cap layout with electrode positions in the extended 10/20 system. Available electrode positions are colored orange. The common ground/reference electrode is colored yellow. The ECG electrodes are colored red, **e** cap equipped with foam pads for comfort

In this work, we used the second version of a reference layer cap prototype, developed by GUGER TECHNOLOGIES OG, Austria (patents pending). This prototype cap offers the opportunity of dedicated reference recordings from a separate layer. The new cap version has Ag/AgCl sinter-pellets as electrode contact areas instead of pure Ag. For a description and an evaluation of the first version please see (Steyrl et al. 2015, 2017). A rendering of the cap is depicted in Fig. 1a and see Fig. 1b for a photo of the new cap version. The cap size is optimized for a head circumference of about 58 cm. However, the cap is flexible enough for head circumferences between 56 and 58 cm. To use this cap with larger heads is not recommended, because in that case the cap can cause pain due to high contact pressure. The cap is equipped with 29 double-layer EEG electrode pairs, a common ground/reference electrode, and connectors for two additional self-adhesive MRI compatible electrocardiogram (ECG) electrodes at the participants back. Each double-layer EEG electrode is made of a pair of Ag/AgCl sinter-pellets with a diameter of approximately 2 mm and a thickness of approximately 1 mm. The pellets are glued with conductive epoxy to both sides of an approximately 1 mm thick printed circuit board (PCB). One pellet connects to the scalp via conductive EEG gel and the other to the reference layer. The PCB with sinter-pellets is fixed into an isolating plastic housing. The whole electrode is about 8 mm thick and has a diameter of approximately 14 mm. For a schematic of a double layer electrode see Fig. 1c. The reference layer itself is a grid of silicon tubes filled with physiological saline solution and is electrically isolated from the scalp, except at the common ground/reference electrode. At this electrode, the scalp is connected to the reference layer to pull them at the same potential. Electrodes are connected to the EEG amplifier via thin copper cables. 5kOhm current limiting resistors were placed between the sinter-pellets and the cables, and additional 5kOhm resistors were placed at the end of the cables before a coupling board connects to the EEG amplifiers via a flat ribbon cable. ECG connectors are equipped with 10kOhm current limiting resistors at the electrodes. The cable length is approximately 50 cm. The electrode arrangement is according to the international 10/20 system and depicted in Fig. 1d. We put foam pads between the occipital EEG electrodes to prevent pain from lying on a few small electrodes, see Fig. 1e. Temperature measurements were carried out during SAR intensive sequences to rule out a harmful heating of the electrodes.

### fMRI Scanner and EEG Recording System

Functional MRI data were acquired at a Siemens Skyra 3.0T (Siemens, Erlangen, Germany) at the MRI-Lab Graz (Austria) using a 20 channel head coil. The helium pump was active and the ventilation was set to the lowest level possible. A standard EPI sequence was implemented (TR = 2250 ms, TE = 28 ms, base resolution = 64, 3.5 × 3.5 × 3.5mm^3^ voxel size, 0.4 mm gap, 36 slices, field of view = 224 × 224). EEG and ECG was recorded with a 64 channel MRI compatible EEG system (BrainAmp MR plus, Brain Products GmbH, Gilching, Germany). The EEG system was positioned inside the borehole at the head end of the MRI scanner on a wooden panel. Cables and amplifiers were fixed with sand bags. All amplifier settings were chosen according to the manufacturer’s recommendations. Hence, the sampling rate was set to 5 kHz, the cut-off frequency of the hardware high pass filter to 0.016 Hz and the cut-off frequency of the hardware low pass filter to 250 Hz. The voltage range was set to ± 16.384 mV, resulting in a resolution of 0.5 μV/bit. The EEG system clock was synchronized with the gradient clock of the MRI scanner via the Brain Products SyncBox device to ensure a highly accurate GA sampling. Sync status has been monitored. BrainVision Recorder (Brain Products GmbH, Gilching, Germany) software version 1.21.0102 was used for EEG data recording. The two ECG channels were treated like EEG channels, hence, EEG settings also apply to ECG recordings. We carefully prepared the electrode skin contact with abrasive electrode gel, but we were not able to control the skin impedances. It would appear that separate ground and reference electrodes must be mandatory to measure impedances with that EEG system.

### Pre-processing Procedure of Outside-MRI-Scanner EEG

**Fig. 2:**
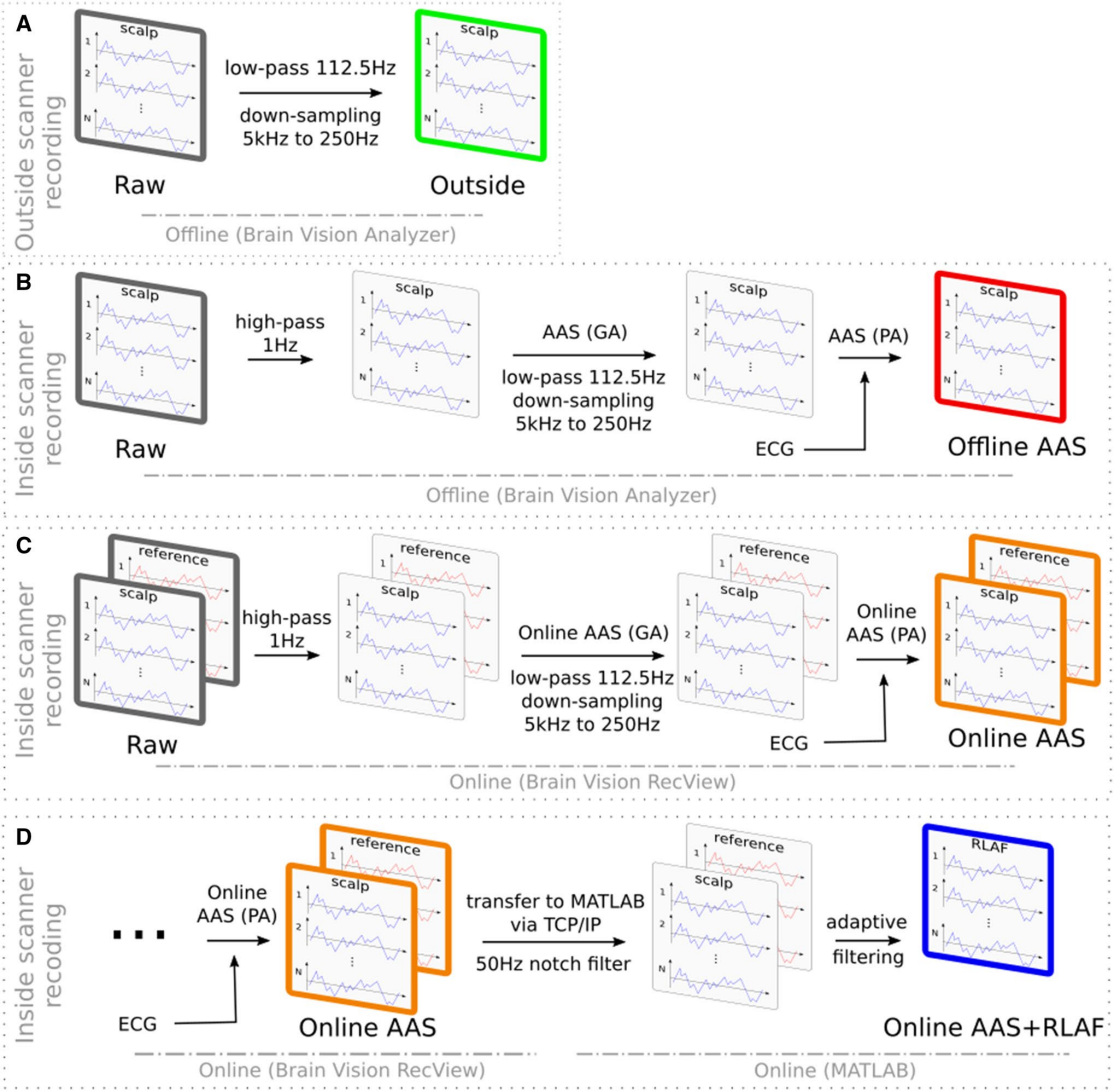
Signal processing overview. **a** Outside-MRI-scanner EEG was low-pass filtered and down-sampled, **b** processing of inside-MRI-scanner EEG to reduce fMRI related artifacts. Average artifact subtraction (AAS) was applied twice. First to reduce the gradient artifact (GA) and second to reduce the pulse artifact (PA), **c** Processing pipeline to reduce fMRI related artifacts online by applying online AAS twice, **d** Additional adaptive filtering step after online AAS to reduce fMRI related artifacts further

After the experiments, outside-MRI-scanner EEG recordings were down-sampled from 5000–250 Hz, using the “Change sampling rate” transformation in the BrainVision Analyzer software (Brain Products GmbH, Gilching, Germany, version 2.1.1.327). That included a 112.5 Hz low-pass anti-aliasing filter with 24 dB/oct damping before the down-sampling. The down-sampling itself is based on spline interpolation. See also Fig. 2a for a summary of the pre-processing procedure. We refer to the EEG after this procedure of outside-MRI-scanner EEG recording and offline EEG pre-processing, when we write of “outside EEG” in upcoming chapters.

### Offline AAS Artifact Reduction Procedure of Inside-MRI-Scanner EEG

BrainVision Analyzer was used to perform artifact reduction offline and included the following steps: (1) Removing signal offsets with a high-pass filter (Butterworth zero phase, cut-off at 1 Hz, 4th order). (2) The next step was GA reduction with AAS as implemented in BrainVision Analyzer. The MRI scanner was sending TTL level triggers during the data recording, to mark new volumes. These markers were used to divide the EEG recordings into GA epochs. GA templates have been calculated separately for each epoch by averaging over 100 adjacent artifact epochs, 50 before and 50 after the current epoch. Subsequently, GA templates were subtracted from EEG recordings and all recordings were down sampled to 250 Hz (low-pass anti-aliasing filter, 112.5 Hz cutoff frequency, 24dB/oct damping). (3) AAS was carried out a second time for PA reduction. To divide the EEG recordings into PA epochs, the semiautomatic R-peak detection mode of the BrainVision Analyzer software was used. In that mode, R-peaks are detected automatically in separate ECG recordings, manually readjusted and subsequently used as markers. As in the GA reduction step, a separate template for subtraction was computed for each PA epoch. 50 adjacent PA epochs, 25 epochs before and 25 epochs after each PA, have been averaged to obtain the PA templates. The procedure is summarized in Fig. 2b. The number of epochs for averaging is a crucial parameter in AAS. It determines the adaptiveness of AAS templates as well as the EEG residuals in the AAS templates. Unfortunately, no gold standard has emerged yet for determining the number of epochs. We base our choice on the following argument: In one of the original papers on AAS (Allen et al. 2000), the aim was to obtain a clean artifact template, in which small events in the EEG are not covered by EEG residuals. They authors assumed that small EEG events have an amplitude of 10 µV and large EEG events have an amplitude of 250 µV, which leads to the use of 25 epochs (Allen et al. 2000). Beside the events argument, using 25 epochs implies that the RMS amplitude of the residual EEG in the template is reduced to 20% of the original RMS amplitude of the EEG, since the RMS amplitude is reduced by a factor of ${\sqrt {number\;of\;epochs} }$. Our goal was to at least maintain that level of residual EEG in two subsequent applications of AAS. Therefore, a reduction to 14% of the original RMS amplitude is necessary in each single step to maintain an overall reduction to 20%. 50 epochs for averaging are necessary to achieve that reduction to 14% and was therefore our choice for the minimum number of epochs. We name the EEG after this procedure of inside-MRI-scanner EEG recording and subsequent offline AAS, as “offline AAS EEG” throughout this work.

### Online AAS Artifact Reduction Procedure of Inside-MRI-Scanner EEG

Inside-MRI-scanner EEG recordings were stored with BrainVision Recorder and were simultaneously sent to BrainVision RecView with the remote data access option of the BrainVision Recorder. Online artifact reduction in RecView included the following steps: (1) High-pass filtering to remove offsets (Butterworth filter, 1 Hz cut-off frequency, 24 dB/oct damping). (2) Online GA reduction with AAS. The TR was used to divide the past EEG into artifact epochs. The first 10 epochs per channel were averaged to compute initial individual GA templates. New epochs were added to the templates if the correlation of the new epoch with the current template exceeded a predefined threshold of 0.975. Subsequently, the current templates were subtracted online from the artifact afflicted EEG. (3) Subsequently, the EEG was down-sampled to 250 Hz (Butterworth low-pass anti-aliasing filter, 112.5 Hz cutoff, 24 dB/oct damping). (4) PAs were tackled with online AAS too. The past EEG was divided into epochs of PAs via online R-peak detection. Online R-peak detection in RecView is based on a template correlation approach. The method searches for a prototypical ECG epoch and subsequently compares it with the ongoing EEG. If certain thresholds are exceeded an epoch is found (settings: minimal pulse period 650 ms, minimal correlation 0.6, minimal amplitude 0.6, maximal amplitude 1.2). Separate PA templates were computed per channel by averaging over the last 50 PA epochs of the respective channel. The current templates were subtracted online from the artifact afflicted EEG. For an overview of this procedure see Fig. 2c. It can be assumed that this online artifact reduction procedure has a maximum delay of 150 ms. It takes 80–100 ms until the EEG data are available in RecView, including the hardware delay of the EEG system, transport of the EEG data via USB and the delay of the BrainVision Recorder software. The actual online artifact reduction in RecView is carried out sample-by-sample and hence, only a small additional delay is added. We assume that this delay is below 50 ms. We abbreviate the EEG after this artifact reduction procedure of inside-MRI-scanner recording and online AAS, with “online AAS EEG” in the following chapters.

### Online AAS + RLAF Artifact Reduction Procedure of Inside-MRI-Scanner EEG

In accordance with previous works, we implemented the adaptive filtering as an additional processing step after GA and PA reduction with AAS (Chowdhury et al. 2014; Steyrl et al. 2017). Online AAS artifact reduction was carried out in BrainVision RecView (see description above). Subsequently, EEG data were transmitted to MATLAB (Mathworks Inc., Natick, MA, USA, Version 2012b) via the BrainVision RecView BCI2000 bridge. This bridge opens a TCP/IP server and the data can be received with any TCP/IP client. Brain Products recommends the pnet TCP/IP client from the TCP/UDP/IP Toolbox for receiving the data in MATLAB. Brain Products provide sample code on their homepage on how to use pnet. In MATLAB, the EEG data were adaptively filtered. The adaptive filtering was directly implemented in MATLAB with the following equations,

1 $${{\text{Subtraction step}}\quad eeg{{\left( n \right)}_{adaptive}}=eeg\left( n \right) - weight\left( n \right) \cdot ref\left( n \right)}$$

2 $${{\text{Weight update step}}\quad weight\left( {n+{\text{1}}} \right)=weight\left( n \right)+step \cdot eeg{{\left( n \right)}_{adaptive}} \cdot ref\left( n \right)}$$

where “n” is the current time sample, “eeg” is the signal of a scalp electrode, “ref” is the signal of the respective reference electrode, “weight” is the respective scaling factor, which we initialized with 1, and “eeg_adaptive_” is the adaptively filtered EEG. “weight” can change its value over time, whereas “step” defines the speed of change. Finding a suitable value for “step” is a trade-off between speed of adaptation (large value) and preventing over-fitting (small value). Based on our experience, we choose a rather small value for “step” of 8 × 10e−7. Our implementation establishes first order models, hence the reference signals are scaled, but no bandwidth limiting filters are learned. The procedure is depicted in Fig. 2d. From here on we term the EEG after this procedure of inside-MRI-scanner recording and online AAS combined with online RLAF as “online AAS + RLAF EEG”.

### Analysis and Performance Metrics

After a visual inspection of an EEG example, we analyze two very common EEG phenomena that were already used as performance criteria for artifact reduction methods in other publications (Chowdhury et al. 2014; Vanderperren et al. 2010). Namely, alpha rhythm amplitude changes and evoked potentials (EPs).

### Alpha Rhythm Amplitude Changes

Oscillatory EEG components often show a brain activity related relative difference in their amplitude compared to a baseline. A prominent example is the occipital alpha rhythm. The amplitude at occipital EEG electrodes rises when one closes his/her eyes. The typical frequency range of that rise is 8–13 Hz. To visualize the amplitude changes, we computed spectra for the opened eyes period and the closed eyes period of the experiment respectively (Welch approach, window length 5 s, overlap 50%). We report the average spectra over the occipital channels (P3, Pz, P4, POz, O1, O2) separate for each participant.

To obtain a performance metric that describes the amplitude change of the alpha rhythm, we calculated the ratios of alpha amplitude between closed and opened eyes with the following equation

3 $${rati{o_\alpha }=\frac{{{A_{close{\text{8}} - {\text{13}}Hz}}}}{{{A_{open{\text{8}} - {\text{13}}Hz}}}}}$$

in which A_close8−13Hz_ is the amplitude during the closed eyes period and A_open8−13Hz_ is the amplitude during the opened eyes period. We report the average of the alpha amplitude ratio over occipital EEG channels (P3, Pz, P4, POz, O1, O2) separate for each participant.

Artifacts or noise in the EEG can cover the amplitude change. Hence, one expects that clean EEG shows a higher alpha amplitude ratio than artifact afflicted EEG. This is generally the case, however, the ratio metric can be distorted by artifacts that (1) have the same frequency range and (2) change with closed and opened eyes. This may apply to PAs. Their frequency range is overlapping with the alpha rhythm and if the PA detection rate is different between opened eyes and closed eyes, then omitted PA artifacts distort the alpha ratio metric. One can avoid this problem in offline PA reduction with AAS, since it is possible to manually search for omitted PAs and to mark them for PA reduction. However, it becomes a problem in online AAS, where a manual intervention is not possible. Therefore, we analyzed the PA detection rate in the online EEG data, and computed the percentage of detected PAs during opened eyes and closed eyes separately for each participant.

With regard to the alpha amplitude ratio metric, it is important to asses its topological distribution. We show the spatial distribution of the metric in separate topo-plots for each participant.

### Visual Evoked Potentials

Evoked potentials are often investigated with respect to their amplitude. We computed the average visual evoked potential (VEPs) of each participant for all different artifact reduction procedures. The depicted channels were selected by the highest outside EEG VEP amplitude of the respective participant.

The VEP signal-to-noise-ratio (SNR) and the similarity of single VEPs to the respective mean VEP are important metrics to quantify VEP quality. We calculated both metrics. The SNR was calculated for each EEG channel separately using

4 $${VEPSN{R_{db}}={\text{20}} \cdot {\text{lo}}{{\text{g}}_{{\text{10}}}}\left( {\frac{{{A_{signal}}}}{{{A_{noise}}}}} \right)}$$

where VEP SNR_db_ is the signal-to-noise-ratio in dB, A_signal_ is the amplitude of the signal, and A_noise_ is the amplitude of the noise. We defined the signal amplitude (A_signal_) as the peak-to-peak amplitude of the first and the second peak in the average VEP. Average VEPs were calculated by averaging band-limited (1–15 Hz) EEG over the VEP trials of the respective EEG channel. We defined the noise amplitude (A_noise_) as the root-mean-square (RMS) amplitude of the band-limited (1–15 Hz) plus-minus (±) reference of the EEG signal of the respective EEG channel (Schimmel 1967). For the (±) reference, odd and even VEPs were averaged separately and subsequently, the average odd VEP was subtracted from the average even VEP. This difference is an estimator of the residual noise in the EEG (Schimmel 1967). The RMS amplitudes of A_signal_ and A_noise_ and therefore the SNR too, are dependent on the bandwidth of the EEG. A smaller bandwidth implies a smaller RMS amplitude and hence a higher SNR, as long as the EP amplitude stays constant. However, the choice of the bandwidth is not crucial as long as it is the same for all calculations, since our intention is to unveil relative differences between the methods. We report the average SNR over occipital EEG channels (POz, O1, O2) separately for each participant.

The root-mean-square (RMS) distance of single VEPs to the average VEP measures the similarity of single VEPs to the respective average VEP. This similarity to the average VEP is equivalent to the variability of single VEPs. The variability has two causes: noise in EEG and the inherent variability of VEPs. One cannot separate these two. However, offline AAS EEG, online AAS EEG and online AAS + RLAF EEG used the same raw EEG data, hence, the underlying inherent VEP variability was the same. Which means that a variability reduction was caused by the artifact reduction method that either reduces the noise in EEG or the inherent VEP variability, or both. It is important to keep in mind, that comparing the RMS distances of inside MRI scanner recordings with outside EEG is problematic since changes in distance could be caused by differences in the inherent VEP variability. RMS distances were normalized to the amplitude of the respective average VEP, since RMS distances are dependent on the absolute signal amplitudes. The distances were calculated per participant and per EEG channel with

5 $${RMS\;distanc{e_j}=\sqrt {\frac{{\text{1}}}{N}\sum\limits_{{n={\text{1}}}}^{N} {{{\left( {avgVEP\left( n \right) - VE{P_j}\left( n \right)} \right)}^{\text{2}}}} } }$$

6 $${NRMS\;distance=\frac{{avgRMS\;distance}}{{VE{P_{amplitude}}}}}$$

where NRMS distance is the average RMS distance divided by the amplitude of the respective average VEP. The “RMS distance” of the jth VEP to the average VEP was calculated using Eq. (5), where “n” is the nth time sample and “N” is the total number of time samples of the EEG data epochs. EEG data epochs had a length of half a second. We report the average NRMS distance over occipital EEG channels (POz, O1, O2) separate for each participant.

## Results

### EEG Example

**Fig. 3:**
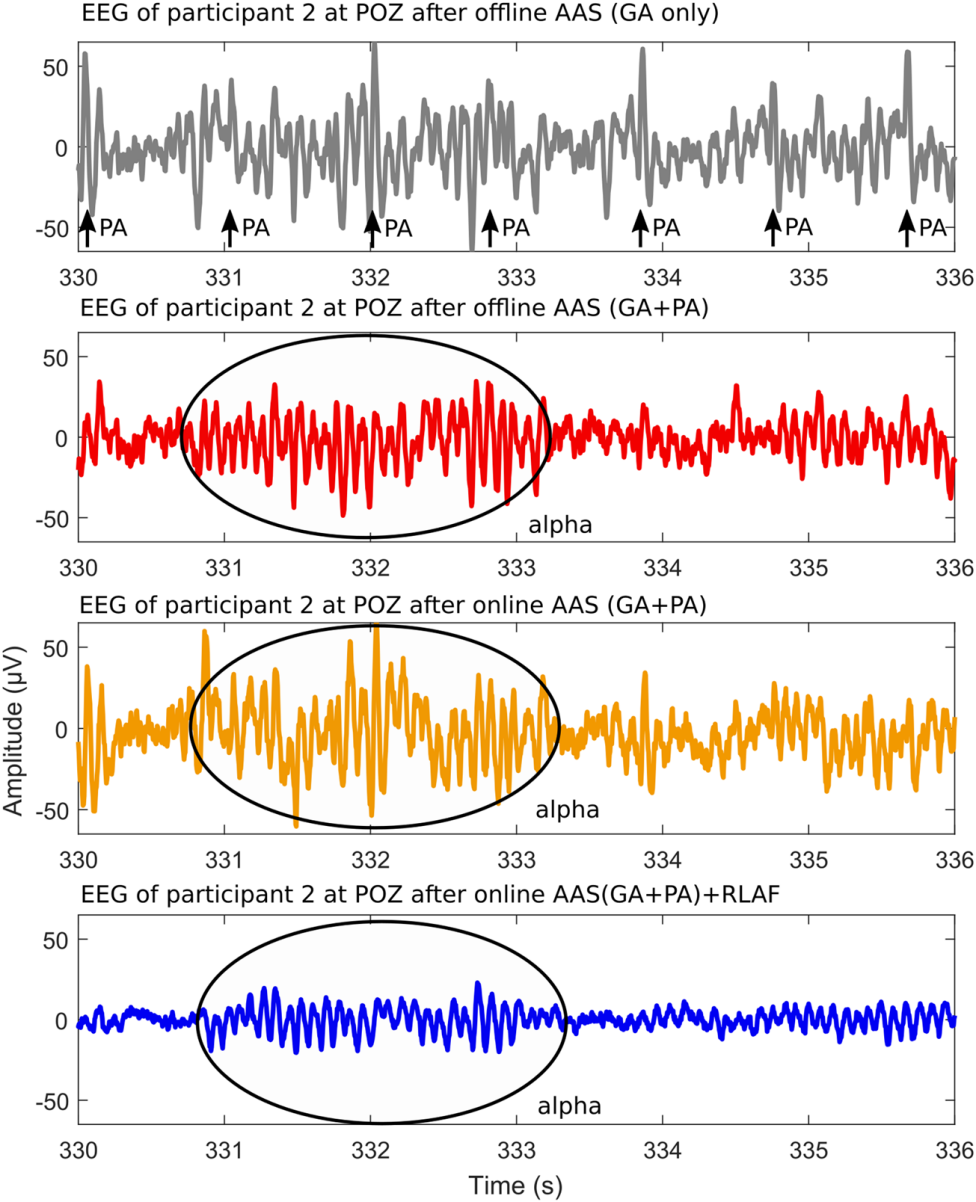
Six second EEG example of participant 3 at electrode POZ, recorded inside the MRI scanner during the closed eyes part of the experiment, 330 to 336 seconds after the start of the paradigm. Pronounced alpha rhythm activity is highlighted. Upper row: EEG after offline average artifact subtraction (AAS) of the GA. Arrows mark pulse artifacts. Second row: EEG after offline AAS of the GA and the PA. Third row: EEG after online AAS (GA+PA). Bottom row: EEG after online AAS (GA+PA) and subsequent online reference layer adaptive filtering (RLAF)

Figure 3 shows a representative example of what EEG of simultaneous EEG-fMRI looks like after the different artifact reduction procedures. The example was taken from participant 3 at electrode POZ and covers the time from 330 to 336 s after starting the paradigm, hence, from the closed eyes part of the experiment. EEG after offline AAS(GA) is depicted in the upper row. GAs were removed and are no lonfer visible, but PAs are clearly identifiable. Maximum PA amplitudes are higher than the usual amplitudes of the EEG. The remaining three rows depict EEG after PA reduction procedures. All procedures are effective to some extent. PA residuals are noticeable after online AAS (GA + PA) e.g. PA residual at second 334. PA residuals are less present after offline AAS (GA + PA) and are hardly noticeable after online AAS + RLAF. The EEG example includes a period of increased alpha activity, which is highlighted in Fig. 3. The period is visible after any of the three artifact reduction procedures. EEG amplitudes differ between the artifact reduction procedures. Highest amplitudes are usually present in online AAS EEG, and smallest amplitudes in online AAS(GA + PA) + RLAF EEG.

### Alpha Rhythm Amplitude Changes

**Fig. 4:**
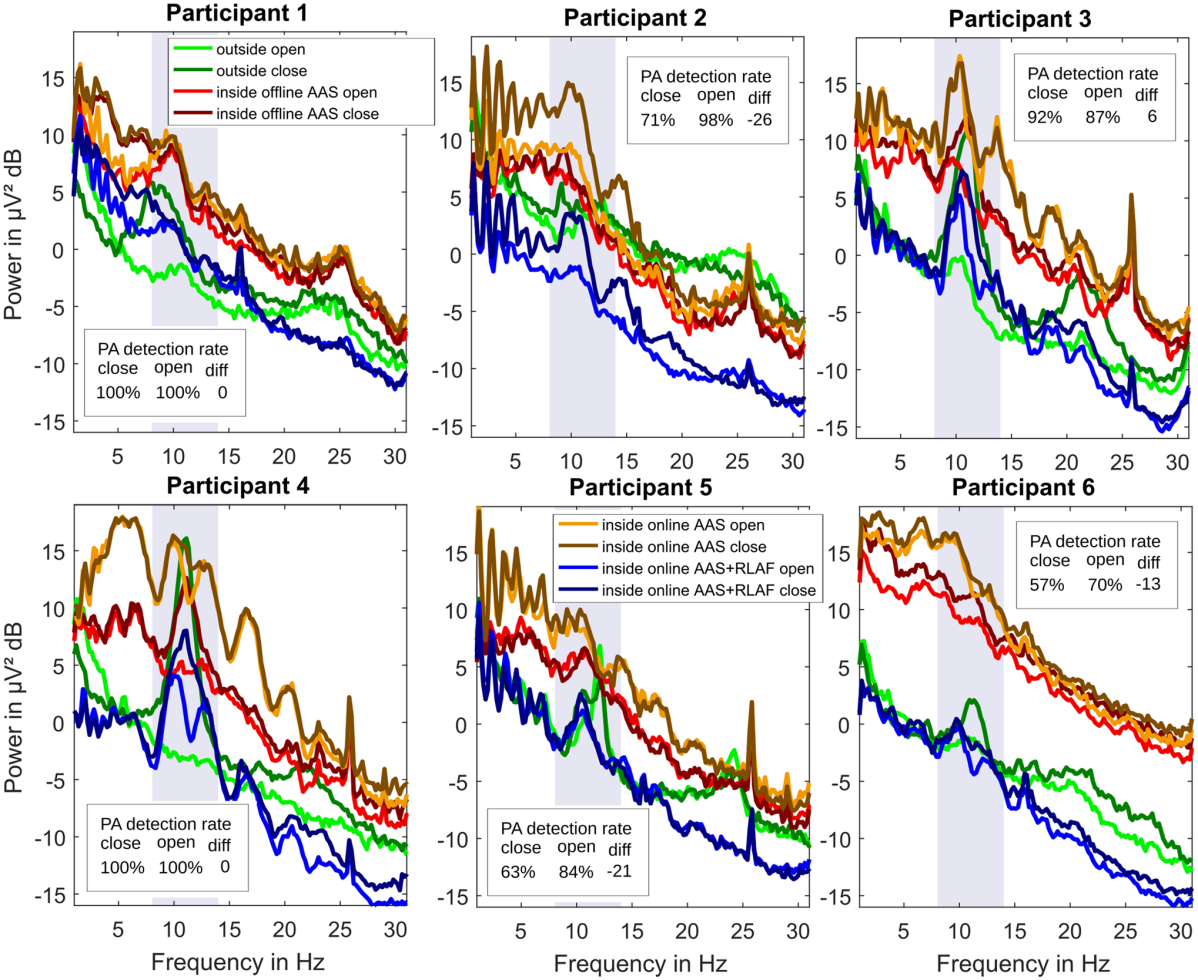
Per participant individual average spectra of EEG at occipital electrode positions (P3, Pz, P4, POz, O1, O2) for opened eyes and closed eyes after different pre-processing and artifact reduction methods (average artifact subtraction AAS, reference layer adaptive filtering RLAF). In boxes, pulse artifact (PA) detection rates of online AAS relative to the pulse artifact detection of offline AAS, separately for opened eyes and closed eyes and the respective difference. The 8–13 Hz frequency range is highlighted

Figure 4 presents spectra in the frequency range of 1–30 Hz, the alpha range of 8–13 Hz is highlighted. In the spectra of outside EEG, a clear alpha peak with closed eyes and a smaller or no alpha peak with opened eyes are the expected results, but the characteristics of the participants’ alpha peaks vary.

During closed eyes sessions, the alpha peaks vary in terms of magnitude (e.g. factor of 4 between participants 4 and 6) and latitude (factor of 5 between participants 2 and 4). Nevertheless, the alpha peak, at least a small one, is recognizable for all six participants in the outside EEG during closed eyes sessions. This is not the case for inside the scanner EEG. In offline AAS EEG, distinct alpha peaks are hardly noticeable in participants 5 and 6 and are very small in participants 1 and 2. Only participants 3 and 4 show clear alpha peaks. In online AAS EEG, participants 2, 3 and 4 all appear to have a distinct alpha peak. The alpha peak is hardly noticeable in EEG of participants 1, 4, and 5. In online AAS + RLAF EEG, it seems that there are distinct alpha peaks in EEG of participants 2, 3, 4, and 5, while the alpha peak is hardly noticeable for participants 1 and 6.

During opened eyes, only participant 5 shows the alpha peak in the outside EEG. Small alpha peaks are noticeable in participants 1, 2, 3, and 6. No alpha peak is noticeable in participant 4. Again the results are different in inside the scanner EEG. In offline AAS EEG, small alpha peaks are noticeable in participants 1, 3, and 5. In the other participants, almost no alpha peak is present. In online AAS EEG, participants 1, 3, and 4 seems to have a distinct alpha peak. In participants 2, 5, and 6, an alpha peak is scarcely noticeable. In online AAS + RLAF EEG, it appears that there are distinct alpha peaks in EEG of participants 2, 3, 4, and 5. Again, in participants 1 and 6 there is almost no alpha peak noticeable.

Alpha rhythm amplitude changes between closed eyes and opened eyes are also different in terms of artifact reduction procedures and participants. Table 1 lists the alpha amplitude ratios for all participants and all artifact reduction procedures. The outside EEG alpha ratios are the highest among methods in participants 1, 3, 4, and 6, second-highest in participant 5 and they are third-highest in participant 2. The offline AAS EEG alpha ratios are second-highest for participants 1 and 6, third-highest for participants 3 and 4, and they are lowest for participants 2 and 5. The online AAS EEG alpha ratios are the highest among methods for participants 2 and 5, and are lowest for participants 1, 3, 4, and 6. Alpha ratios of online AAS + RLAF EEG are second-highest among methods for participants 2, 3, and 4, and they are third-highest for participants 1, 5, and 6.

The online PA detection rate was not stable in all participants. In Fig. 4, the differences in online PA detection rate between opened eyes and closed eyes are noted in extra boxes. Negative differences of − 26, − 21, and − 13 percent points were found in participants 2, 5, and 6. A negative difference implies that the PA detection rate was higher during opened eyes and it is likely that the respective alpha ratio is increased by artifacts that are not reduced. It can be assumed that the increase of the alpha ratio is proportional to the difference in percent points. No differences in the PA detection rate was found in participants 1, and 4. A small positive difference was found in participant 3. A positive difference implies that the PA detection rate was higher during closed eyes and hence it is likely that the alpha ratio is decreased by artifacts that are not reduced.

**Table 1:** Average alpha amplitude ratio of closed eyes to opened eyes at occipital EEG channels (P3, Pz, P4, POZ, O1, O2)

Alpha ratio (AU)	Outside EEG	Inside offline AAS EEG	Inside online AAS EEG	Inside online AAS + RLAF
Participant 1	1.71	1.06	1.01	1.04
Participant 2	1.12	1.10	1.61	1.50
Participant 3	2.53	1.36	1.06	1.39
Participant 4	3.62	1.57	1.09	1.58
Participant 5	1.04	0.96	1.08	1.02
Participant 6	1.27	1.22	1.08	1.12

EEG was recorded outside the scanner (outside) and inside the MRI scanner simultaneously with fMRI. Different artifact reduction procedures were applied to the inside-MRI-scanner EEG. Average artifact subtraction (AAS) was applied to the EEG after the recording (offline) or online during the recording (online). Reference layer adaptive filtering (RLAF) was applied online as an additional step after online AAS. Higher values are better

**Fig. 5:**
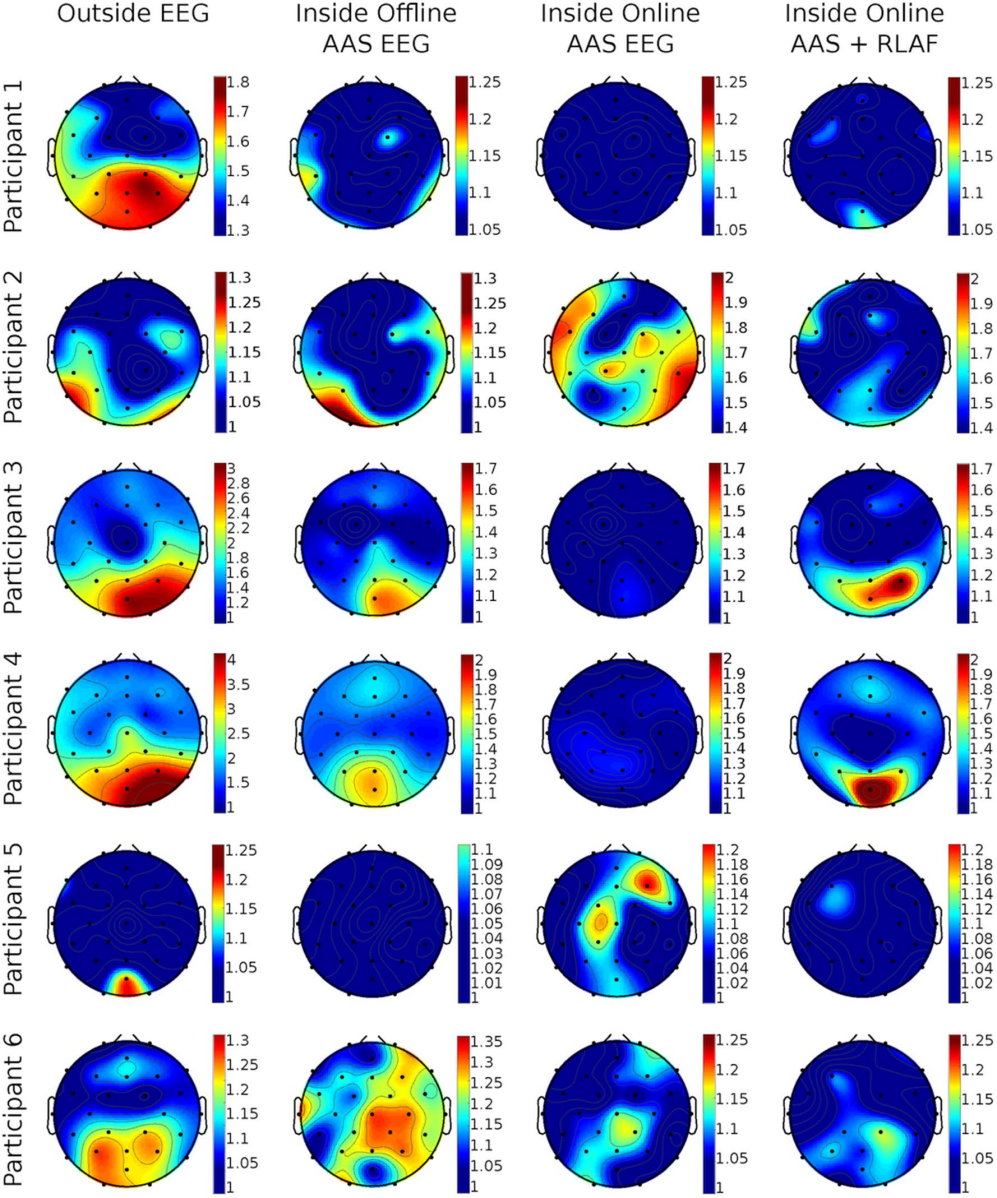
Per participant topological mapping of the respective alpha amplitude ratios (8–13 Hz) after different pre-processing and artifact reduction methods (average artifact subtraction AAS, reference layer adaptive filtering RLAF). Alpha ratios were calculated between closed and opened eyes, hence, higher values imply higher changes. Please note the different scalings

To visualize the topographic distribution of alpha amplitude ratios, we mapped the ratios to 2D electrode positions in Fig. 5. The first column depicts the alpha amplitude ratios of outside EEG for all six participants. As expected the alpha amplitude ratios at occipital electrode positions are commonly larger than those on central or frontal positions. However, differences between participants in ratio sizes and spatial distribution are obvious. Column two shows the alpha ratio topo-plots of offline AAS EEG. The aforementioned pattern is not present in all participants anymore. For example, participant 1 and participant 5 shows only small changes in alpha amplitude between closed and opened eyes and participant 6 shows a pattern where the highest alpha ratios are present in central electrodes. Online AAS EEG alpha ratio topo-plots are presented in in column 3. No participant has the expected pattern of higher ratios at occipital electrodes. For example, participants 2, 5 and 6 have their highest alpha ratios at central or frontal electrodes. The topo-plots of the online AAS + RLAF alpha ratios in the last column shows higher occipital alpha ratios in participants that exhibited almost no changes in alpha amplitude in online AAS EEG (participants 1, 3, 4). In those participants with highest alpha ratios in central or frontal electrodes (participants 2, 5, 6), online AAS + RLAF was able to reduce those ratios. It appears that the topo-plots of online AAS + RLAF are often more similar to the topo-plots of offline AAS EEG than to the topo-plots of online AAS EEG.

### Visual Evoked Potentials

**Fig. 6:**
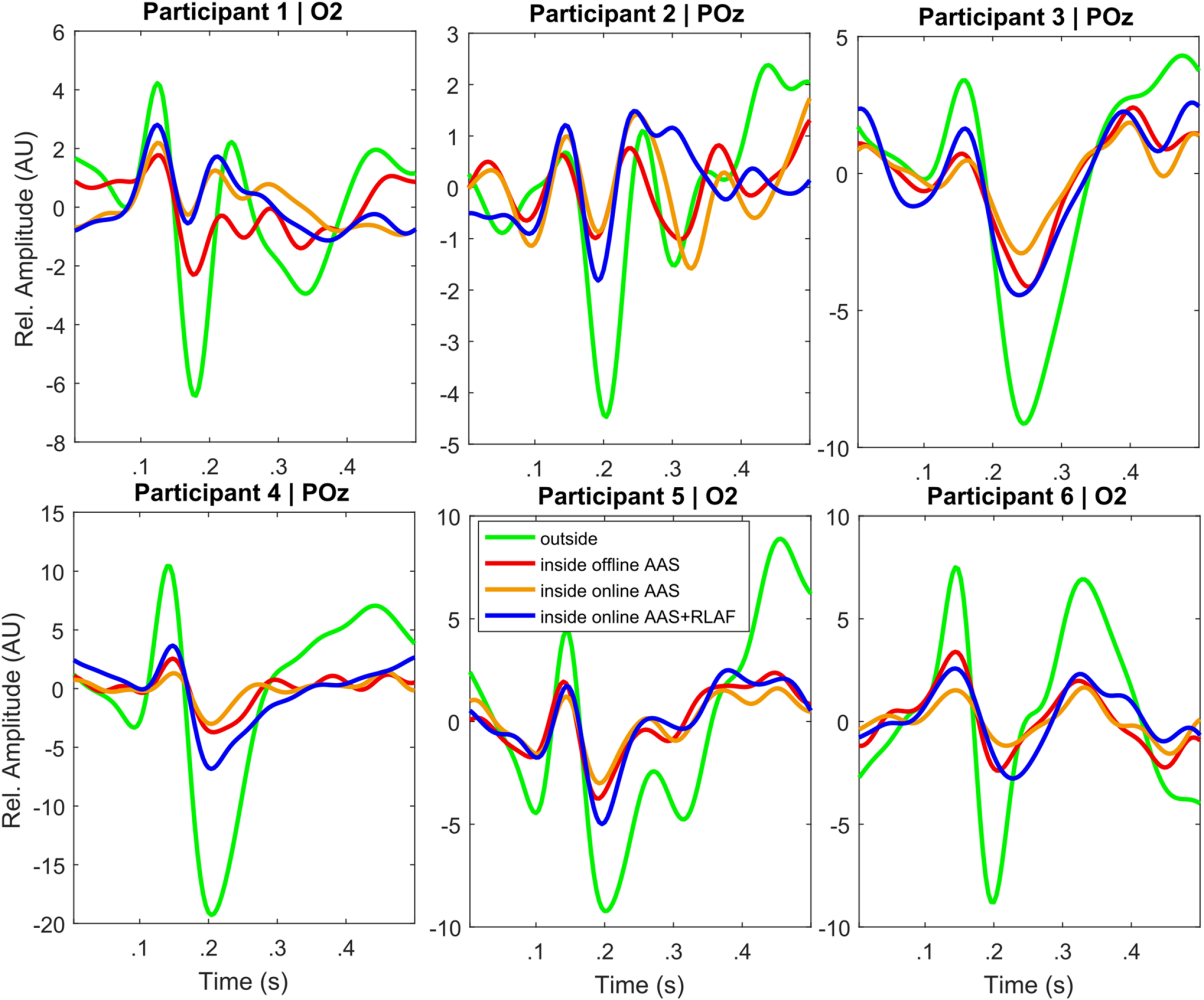
Single participant VEPs for different EEG recording and pre-processing procedures [average artifact subtraction (AAS), reference layer adaptive filtering (RLAF)]. Channels were selected by the highest VEP amplitude of outside EEG. All VEPs were normalized by the RMS amplitudes of the respective (±) reference. Please be aware of the different scaling of the y axis

Figure 6 shows single participant VEPs for all different pre-processing and artifact reduction procedures. The respective channel was selected because of the highest outside EEG VEP amplitude of the participant. The VEP amplitudes were normalized by the RMS noise amplitudes of the (±) reference. VEP amplitudes are highest in outside EEG among all 6 participants. In offline AAS EEG VEP amplitudes are second-highest in participants 1 and 6 and third-highest in participants 3, 4, and 5. In online AAS EEG, VEP amplitudes are third-highest in participant 2. In online AAS + RLAF EEG, VEP amplitude are second-highest in participants 2, 3, 4, and 5, and they are third-highest in participants 1 and 6. In all 6 participants, VEP amplitudes in online AAS + RLAF EEG are higher than in online AAS EEG.

For VEPs, the signal-to-noise-ratio describes the ratio of the VEP amplitude to the respective residual noise amplitude, hence, the distinctness of the VEPs. Table 2 collects the SNRs of all pre-processing and artifact reduction procedures and all participants. All SNRs are positive, indicating that VEP amplitudes are higher than the residual noise. In outside EEG, the VEP SNR is highest for all 6 participants. In offline AAS EEG, the SNR is second-highest in participants 1, 4, 5, and 6 and third-highest in participants 2 and 3. In online AAS EEG, the SNR is lowest for all 6 participants. In online AAS + RLAF EEG, the SNR is second-highest in participants 2, 3, and 5 and third-highest in participants 1, 4, and 6. A pattern is noticeable. Highest SNRs in outside EEG, second-highest SNRs in offline EEG or in online AAS + RLAF EEG with small differences only, and lowest SNRs in online AAS EEG. SNRs in online AAS + RLAF EEG are higher than in online AAS EEG for each participant.

**Table 2:** Average signal-to-noise-ratio (SNR) of visually evoked potentials (VEP) at occipital EEG channels (POZ, O1, O2)

SNR in dB	Outside EEG	Inside offline AAS EEG	Inside online AAS EEG	Inside online AAS + RLAF
Participant 1	20.7	10.2	6.9	8.6
Participant 2	13.7	5.7	3.6	6.6
Participant 3	20.1	15.5	12.4	17.4
Participant 4	24.2	14.7	8.9	14.5
Participant 5	23.1	15.5	12.0	15.5
Participant 6	23.6	11.5	7.3	11.1

EEG was recorded outside the scanner (outside) and inside the MRI scanner simultaneously with fMRI. Different artifact reduction procedures were applied to the inside-MRI-scanner EEG. Average artifact subtraction (AAS) was applied after the recording (offline) or online during the recording (online). Reference layer adaptive filtering (RLAF) was applied online as an additional step after online AAS. Higher values are better

Another performance metric that describes VEP quality is VEP variability. This criterion describes how similar single VEPs are to the respective average VEP. Similarity is measured with the normalized root-mean-square distance of single VEPs to the respective average VEP. Table 3 presents the average VEP distance (NRMS distance) at occipital EEG channels for all pre-processing and artifact reduction procedures and all participants. A smaller value denotes a smaller distance, hence a lower variability or a higher similarity. Offline EEG showed the lowest NRMS distance, hence, VEP variability in all 6 participants. Offline AAS EEG showed the second-lowest variability in participants 1 and 5 and the third-lowest in participants 2, 3, 4, and 6. Online AAS EEG showed the highest variability in all single participants. Online AAS + RLAF EEG showed the second-lowest variability in participants 2, 3, 4, and 6, and the third-lowest in participants 1 and 5. The same pattern as with VEP SNR is visible. Lowest variability in outside EEG, second-lowest variability in offline EEG or in online AAS + RLAF EEG, and highest variability in online AAS EEG. Variability in online AAS + RLAF EEG is lower than in online AAS EEG for each participant.

**Table 3:** Average normalized root-mean-square-distances (NRMSD) of single visual evoked potentials (VEP) to the respective mean VEP at occipital EEG channels (POZ, O1, O2)

NRMSD AU	Outside EEG	Inside offline AAS EEG	Inside online AAS EEG	Inside online AAS + RLAF
Participant 1	1.2	3.1	4.5	3.8
Participant 2	2.8	7.0	9.5	5.3
Participant 3	0.7	2.3	3.1	2.1
Participant 4	0.8	2.0	3.5	1.9
Participant 5	0.9	2.2	3.6	2.4
Participant 6	0.7	6.9	9.6	3.0

EEG was recorded outside the MRI scanner (outside) and inside the MRI scanner simultaneously with fMRI. Different artifact reduction procedures were applied to the inside-MRI-scanner recorded EEG. Average artifact subtraction (AAS) was applied after the recording (offline) or online during the recording (online). Reference layer adaptive filtering (RLAF) was applied online as an additional step after online AAS. Smaller values are better

**Fig. 7:**
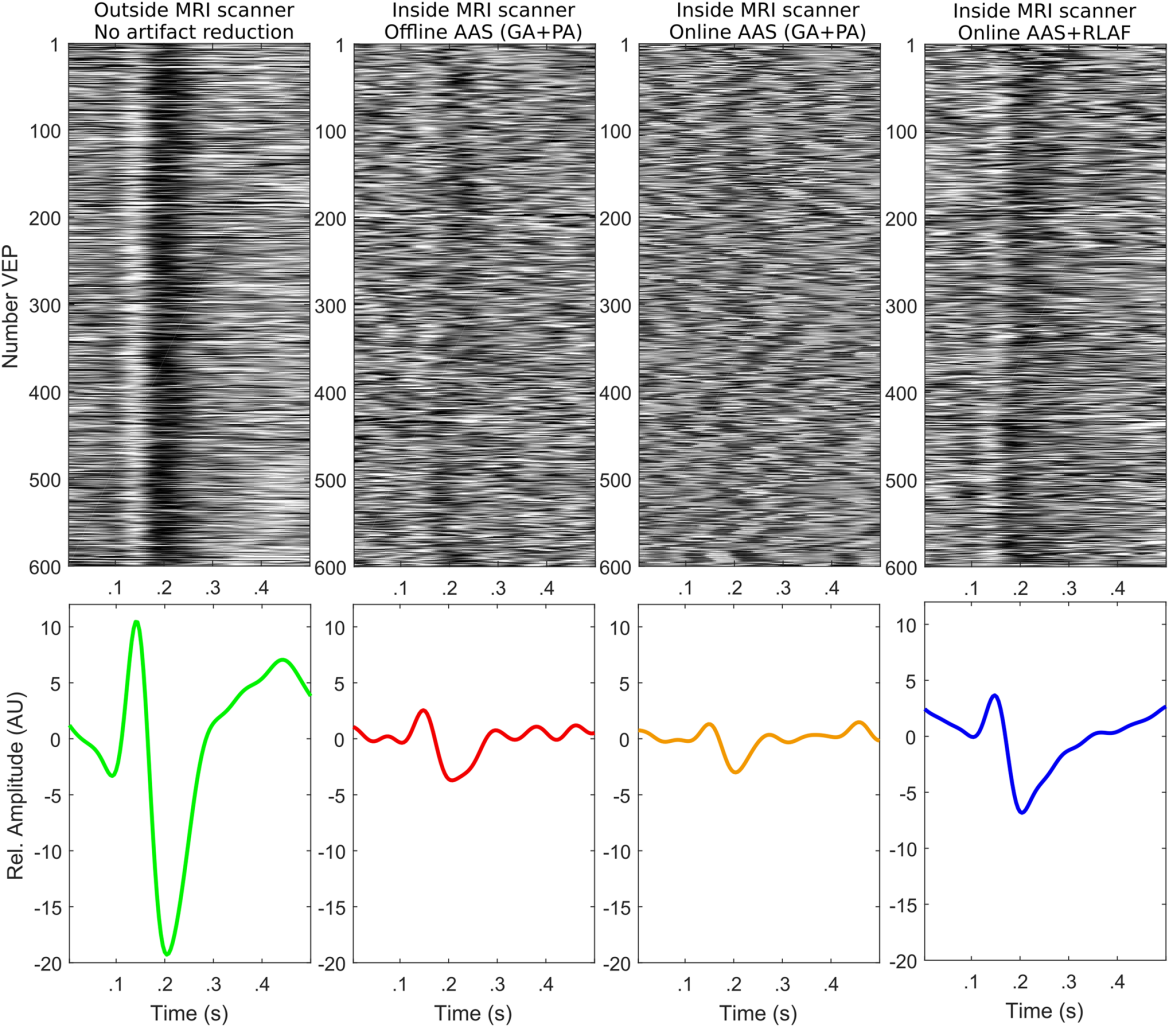
Representative examples of visually evoked potentials (VEPs) for different pre-processing and artifact reduction procedures (average artifact subtraction AAS, reference layer adaptive filtering RLAF). Examples are from participant 4 at electrode POZ. Upper row: single VEPs at electrode POZ (1–15 Hz). Bottom row: average VEPs at electrode POZ scaled to the EEG noise amplitude. Hence, VEP amplitude divided by the root-mean-square value of the (±) reference

We exemplify VEP similarity in Fig. 7. The upper row depicts the single VEPs of participant 4 at electrode POZ for all pre-processing and artifact reduction procedures. In our example, single VEPs are most distinctive in outside EEG, followed by online AAS + RLAF EEG and offline AAS EEG. In online AAS EEG, single VEPs are hardly noticeable. These differences are also present in the average VEPs in the bottom row of Fig. 7. The peak-to-peak amplitude of the normalized average VEP is highest in outside EEG, followed by online AAS + RLAF EEG and offline AAS EEG. It is lowest in online AAS EEG.

## Discussion

We start the discussion with a comparison of online AAS artifact reduction with its offline variant, hence a diagnosis of the state-of-the-art. Subsequently, we discuss improved EEG quality through the additional RLAF step after online AAS in the main part of the discussion. Thereafter, we comment on EEG quality differences between inside and outside the MRI scanner recorded EEG, we share our experience with the new EEG-cap prototype and finally, we discuss limitations of this work.

### Current State: Offline AAS Versus Online AAS

Effective artifact reduction in EEG of simultaneous EEG-fMRI is hard to achieve in general. It is even harder to achieve, when the artifact reduction has to be performed online. We have included offline AAS in this work to illustrate the performance differences between offline and online AAS.

In the visual inspection of the EEG example, we found larger artifact residuals in online AAS EEG than in offline AAS EEG. The artifact at second 334 gives a good impression of the difference. Changes in the alpha range showed two different patterns. (1) Participants that showed a stable PA artifact detection rate in online AAS (participants 1, 3, 4) have larger changes in the alpha range of offline AAS EEG than of online AAS EEG. This visual finding is supported by the alpha amplitude ratios and also depicted in the topo-plots, where we find the same pattern. Participant 6 exhibits also larger changes in the alpha range of offline AAS EEG although the PA detection rate between closed and opened eyes was different, however, the topo-plots shows that these are presumably caused by artifacts. (2) Participants with differences in the PA detection rate (participants 2 and 5) show a different pattern. They have smaller changes in the alpha range of offline AAS EEG than of online AAS EEG. These smaller changes do not imply, however, that online AAS performed better than offline AAS in those participants, but mean that omitted PAs had a stronger influence than the change in alpha rhythm. In the context of VEPs, the SNR is higher in offline AAS EEG than in online AAS EEG and the NRMS distance of VEPs is smaller in all participants. That relation is also visible in the VEP similarity example in Fig. 7, where single VEPs are noticeable in offline AAS EEG, but not in online AAS EEG.

Both offline and online AAS, are based on the same idea, namely to create an artifact template through averaging over adjacent artifact epochs and to subtract the template from the EEG to remove the artifact. However, offline and online AAS naturally differ due to the available EEG data in the respective technique. In offline AAS, it is possible to consider future artifact epochs to construct templates. Those future artifact epochs are also useful to detect PA epoch onsets and it is possible to adjust epoch onsets manually. This is not possible in online AAS. We have been observing periods of up to a minute without working PA epoch detection in online AAS EEG of participant 6. Omitted PAs, however, cannot fully explain the performance differences found between offline and online AAS, since the online PA detection worked almost perfectly in participants 1 and 4 and the online AAS performance was still lower than the offline AAS performance. Hence, the difference in artifact template construction must also be responsible.

In summary, a clear pattern is present in our data, namely that online AAS is less effective than offline AAS.

### Online AAS EEG Quality Improvement Through Additional Online RLAF

In our last work on EEG artifact reduction in simultaneous EEG-fMRI, we compared reference layer adaptive filtering with (1) its direct predecessor, which is termed reference layer artifact subtraction (RLAS), and with (2) plain AAS as the assumed most common artifact reduction technique (Steyrl et al. 2017). Among these techniques, we found that RLAF is the most effective one, if RLAF is applied as an additional signal processing step after AAS. This result concerning the order of technique combination has also been reported by Chowdhury et al. for RLAS (Chowdhury et al. 2014). Due to this experience and due to the need for higher EEG quality in online artifact reduction, we extended RLAF to be applicable online.

The EEG example illustrates the effect of online RLAF on EEG. Three main effects are visible: (1) Generally, smaller amplitudes are an obvious effect of RLAF. Peak-to-peak amplitudes dropped from approximately ± 50 to ± 0 µV. This effect comes from the adaptive subtraction and was already reported by Chowdhury et al. in their work about RLAS and in our last work on RLAF (Chowdhury et al. 2014; Steyrl et al. 2017). (2) Residual artifacts are hardly identifiable in online AAS + RLAF EEG, but are visible in online AAS EEG. (3) The period of enhanced alpha activity is present in both online AAS EEG and online AAS + RLAF EEG.

In the spectra, we once again found two patterns of amplitude changes in the alpha range and they are the same as for offline AAS versus online AAS. (1) Participants with a stable PA artifact detection rate in online AAS (participants 1, 3, 4) exhibit larger changes in the alpha range of online AAS + RLAF EEG than of online AAS EEG. Participant 6 again showed the same pattern. (2) Participants with an unstable PA detection rate (participants 2 and 5) again showed the opposite pattern, hence smaller changes in the alpha range of online AAS + RLAF EEG than of online AAS EEG. This visual finding is reflected in the alpha amplitude ratios in Table 1 too. Both the larger and smaller alpha amplitude ratio results, however, imply that RLAF is removing residual artifacts and is improving the EEG quality for those participants. RLAF shows a different behavior because the main cause of the amplitude changes differs in those participants. In participants 1, 3, 4, and 6, the PA detection ratio between closed and opened eyes was stable or relatively stable and the main cause for amplitude changes was thus the alpha rhythm. RLAF was able to sharpen that amplitude change by removing residual artifacts and therefore, alpha amplitude ratios are larger in online AAS + RLAF EEG than in online AAS EEG for those participants. On the other hand, in participants 2 and 5, the PA detection ratio between closed and opened eyes was not stable, and hence omitted PAs were the main cause for amplitude changes. RLAF is reducing these artifacts, and hence the alpha amplitude ratios are smaller in online AAS + RLAF EEG than in online AAS EEG for those participants. These considerations are supported by the offline AAS results. Alpha amplitude ratios of offline AAS EEG are not afflicted by the stability of the PA detection rate, since we manually corrected omitted PAs. The following pattern can be seen: RLAF improves the alpha amplitude ratio towards the alpha ratios of offline AAS for all participants, with improvements from 3 to 45%.

A change in alpha amplitude at occipital EEG channels between closed and opened eyes is expected in the topo-plots, hence a larger alpha amplitude ratio at these channels. Such patterns are hardly noticeable, however, in online AAS EEG. Nonetheless, they are visible in offline AAS EEG, which indicates, that changes actually do occur in alpha amplitudes as expected. In online AAS + RLAF EEG on the other hand, alpha amplitude changes are visible and in single participants even more pronounced than in offline AAS EEG. These patterns are often more similar to the patterns of offline AAS EEG than to the patterns of online AAS EEG and as a result these topo-plots give the impression that online RLAF is able to unveil the alpha amplitude changes from online AAS EEG.

We found a straight-forward pattern in the single participants VEPs. The VEP amplitudes are larger for all participants in online AAS + RLAF EEG than in online AAS EEG, whereby the VEP shapes are hardly changed. The shapes are also similar to outside EEG VEP shapes, but with lower amplitudes.

The VEP SNRs of online AAS + RLAF EEG are also higher in all single participants than the VEP SNRs of online AAS EEG, with SNR gains between 25 and 63%. (V)EP experiments typically require numerous repetitions, since averaging is commonly the method of choice for getting rid of the ongoing EEG and residual artifacts and consequently to make EPs visible. The starting SNR and the number of repetitions define the resulting EP quality, hence the final SNR after averaging. A higher starting SNR makes it possible to reduce the number of repetitions while maintaining a specific (V)EP SNR or it allows for higher (V)EP SNR within the same experiment duration. Both options are greatly welcomed by neuroscientists.

Normalized-root-mean-square-distances of single VEPs are lower in online AAS + RLAF EEG than in online AAS EEG for each single participant, with differences between − 16 and − 44%. The variability reduction is caused by either noise reduction, including artifact residuals, or reduction of the inherent VEP variability, or both. A reduction of the inherent VEP variability implies a loss in VEP signals and is therefore unwanted. However, since VEP shapes are not altered in online AAS + RLAF and VEP SNRs are simultaneously improved, we argue that the VEP signal loss is only minor and that online RLAF is mainly reducing noise and artifacts.

Single VEPs are hardly noticeable in online AAS EEG of Fig. 7. The variability in this EEG is too high. In contrast, single VEPs are visible in online AAS + RLAF EEG, because of the lower variability. It is noteworthy that the bandwidth was the same for both.

Several possible causes are apparent as to why RLAF improves EEG quality over online AAS EEG. (1) RLAF is able to reduce PAs that were omitted by the online PA detection, and hence, were not reduced in AAS. (2) Residual PAs are present after AAS and they mask the EEG. For example, participant 4 had an exceptionally high alpha power ratio, as unveiled with offline AAS. However, this high ratio is not visible in online AAS EEG and for this participant in particular, we observed significant PA residuals over the whole experiment duration, although the PA detection rate was about 100%. RLAF reduced the PA residuals and unveiled the alpha power changes. (3) RLAF reduces other artifacts or residuals of other artifacts too, as long as they are represented in the reference layer of the cap. None of these possible causes alone can explain all of the EEG quality improvements. Hence, we assume that a combination of them is responsible for the observed quality improvement.

Occasionally, online AAS + RLAF can even compete with offline AAS. e.g. in participants 1, 3, and 4, alpha amplitude ratios in online AAS + RLAF EEG are on eye level with ratios in offline AAS EEG. In participants 2, 3, 4, 5, and 6, the VEP SNRs in online AAS + RLAF EEG are on eye level with SNRs in offline AAS EEG. Online AAS + RLAF can keep up with offline AAS even at 3T MRI scanners, also when comparing the example of raw EEG, the alpha power ratio mapping, and the VEP variability.

A practical advantage of the RLAF technique is its low demand on computing power. The complete system consists of (1) the BrainProducts software that takes on the recording and the online AAS, (2) a Matlab script that handles the paradigm control and the adaptive filtering and (3) the communication required between these components. The system was running on a laptop with an Intel Core i7 mobile CPU at 2.4 GHz and 8 GB RAM. Windowing or storing of old data is not required in the online RLAF part, adaptive filtering steps are computed sample-by-sample. Hence, the additional RLAF step only adds a delay of one sample for processing and the delay of the network communication to the artifact reduction process of the BrainProducts system. However, a block processing scheme is also entirely feasible, that would be able to speed up the computation at higher sampling rates.

In summary, our performance metrics document that online RLAF is able to effectively reduce residual MRI related artifacts in online AAS.

### EEG of simultaneous EEG-fMRI compared to EEG from outside the MRI

We did not yet comment on the general EEG quality loss of simultaneous EEG-fMRI compared to EEG that was recorded outside the MRI scanner. Such comparisons have been made already, particularly in the papers of Allen et al. in which they invented the AAS technique (Allen et al. 1998, 2000). However, such a comparison was still missing for the reference layer cap prototype and in addition we are not aware of a comparison between outside EEG and online AAS EEG.

Our performance metrics show that in any terms of comparison, the inside MRI scanner EEG quality never reaches that of outside EEG. The differences are substantial. For example, alpha amplitude ratios are higher in outside EEG than in any EEG of simultaneous EEG-fMRI if the alpha amplitude changes were not caused by artifacts. Other examples are SNRs of VEPs and NRMS distances of VEPs, where we see the same: Simultaneous EEG-fMRI recording comes at the cost of EEG quality. Nevertheless, simultaneous EEG-fMRI enables us to address new research questions about the human brain, which cannot be answered without this combination of techniques. Hence, this gap in EEG quality demonstrated how important new techniques are that improve the quality of inside MRI scanner recorded EEG, such as the one we present here in this work with the RLAF technique.

### Reference Layer Cap Prototype

The old reference layer cap prototype, that was used in our last work on RLAF, became unusable after several applications (Steyrl et al. 2017). The electrode contact areas were made of copper and coated with silver. Unfortunately the abrasive electrode gel removed the silver coating and the underlying copper was revealed. The copper formed a half cell potential with the remaining silver, leading to a high offset voltage that caused permanent saturation at the amplifier. The new reference layer cap prototype overcomes this major drawback by using Ag/AgCl sinter pellets as electrode contact areas. The pellets are about 1 mm thick and as a result can resist the abrasive gel much longer. We did not notice a degradation of the pellets after 20+ (test) measurements. We assume that the durability of the electrodes of the new prototype cap will be similar to standard EEG electrodes. The advantages of the old cap prototype are valid for the new cap too. It is compatible with available EEG amplifier systems, which allows the upgrading for existing systems, its preparation and handling times are similar to standard EEG caps, no additional susceptibility artifacts are noticeable in fMRI recordings, and EEG of reasonable quality became visible after AAS.

### Limitations

It is not possible to compare our results statistically, due to the limited number of participants. Hence, all comparisons imply a numerical difference only. Nevertheless, as described above, our results show very similar patterns in the performance metrics among all participants: (1) online AAS + RLAF superior to online AAS and (2) occasionally at eye level with offline AAS. (3) outside MRI scanner recordings superior to all inside scanner techniques. These patterns were stable among participants, with only two exceptions. The alpha amplitude ratios of participant 2 and 5 were highest in online AAS EEG, and lowest in offline AAS EEG. We assume that the reason for this deviation from the pattern is the unstable detection of PA epoch onsets. The onset detection failed more often during the eyes closed part of the experiment. This was visible in the spectrum as a higher power in lower frequency ranges, including the alpha range. We thus attribute the deviation of participants 2 and 5 to the higher number of PAs that are not reduced in the eyes closed part of the experiment. This behavior of the alpha amplitude ratios demonstrates one weakness of this metric, it is depending on a constant performance of the artifact reduction over the whole experiment duration.

Regarding our choice of the number of epochs for averaging in AAS, it is important to note that another number possibly leads to better results of AAS. We did not optimize that number via e.g. a pre-study.

The online artifact reduction procedure is not of course instantaneous. The maximum delay of online AAS can be assumed to be 150 ms and RLAF adds a marginally delay only. Nevertheless, the overall delay needs to be determined accurately in a future work, since this delay is crucial for experiment design.

Another limitation of this work concerns the inside/outside MRI EEG comparison. It must be noted that although the experiment was the same inside and outside the MRI scanner and the cap stayed in place between the two experiments, the recordings are not necessarily comparable, since the environment parameters changed. For example, the distance to the screen was different inside and outside the scanner, participants were in sitting position outside and in lying position inside and outside it was quiet but inside it was loud. Hence, natural changes of the EEG over time cannot be ruled out as a source of differences, since the order of inside and outside EEG measurements was not randomized.

## Conclusion

EEG quality is generally impaired when simultaneously acquired with fMRI. This impairment is even more pronounced, when artifact reduction techniques have to be performed online. Our results document this behavior for AAS, namely that online AAS is less effective than offline AAS. We extended the technique RLAF from offline to online use in order to improve online artifact reduction. We showed that online AAS + RLAF achieves higher numerical performance in all metrics when compared to online AAS. Further, we demonstrated that online AAS + RLAF is occasionally even comparable with the offline AAS artifact reduction technique at 3T MRI scanners. Based on these results, we believe online RLAF to be an add on technique after AAS, which has the potential to become a very important tool in the field of simultaneous EEG-fMRI and that will allow us to carry out simultaneous EEG-fMRI experiments at a new level of EEG quality.
